# Data to evaluate the usability of an interactive system based on a judgment-based model

**DOI:** 10.1016/j.dib.2022.108418

**Published:** 2022-06-27

**Authors:** Adeleh Asemi, Asefeh Asemi

**Affiliations:** a Department of Software Engineering, Faculty of Computer Science and Information Technology, Universiti Malaya, 50603 Kuala Lumpur, Malaysia; b Doctoral School of Economics, Business, & Informatics, Corvinus University of Budapest, 1093 Budapest, Fovam ter 8., Hungary

**Keywords:** Interactive systems, Usability assessment, Multifactorial assessment, Fuzzy inference system, Human-computer interaction, Active machine learning methods, Interactive systems for people with disabilities

## Abstract

The data presented in this article are related to the published article entitled “A Judgment-Based Model for Usability Evaluating of Interactive Systems Using Fuzzy Multi Factors Evaluation (MFE)” in “*Applied Soft Computing*” [1]. The purpose of data collection in this paper was to integrate a fuzzy multifactorial evaluation (MFE) model based on the judgment of experts in the three fields of ISPD, HCI, and AMLM. Two sets of data were used to conduct this research. One set of data extracted from WoS related to 180 articles published in 2018-2019. The data were extracted by searching the keyword “interactive system” in the “Computer Science” category. The second category of data is related to the opinions of experts. Component factor analysis in “IBM SPSS 25 Statistics” was used to classify the objectives of the interaction system. The collected data were prepared as FIS inputs. A FIS was designed to evaluate usability using a fuzzy toolbox of MATLAB software of Mamdani type. Inputs consisted of four classes of interactive systems and five usability criteria as outputs. One of the inputs as the target of the interactive system was not considered a fuzzy variable. The rest of the inputs and five outputs were considered the fuzzy variable.

## Specifications Table

**Table:** 

Subject	Computer Science: Artificial Intelligence
Specific subject area	Evaluation of interactive systems: Today, extensive research has been done on the evaluation of interactive systems and human-computer interaction and related topics. The research has been done to investigate the extent to which the goals and expectations of the user are met by an interactive system [12].Multifactorial Fuzzy Evaluation (MFE): Fuzzy multifactorial methods are associated with uncertainty and user preferences. They can also be used to assess usability. Fuzzy distance calculation and fuzzy pairwise comparison are two popular methods in MFE [4] & [10]. These methods have also been used in previous research to compare or rank the use factors or usability of different systems [5], [6], [7], [8].
Type of data	ImageMicrosoft Visio Document (.vsd) fileSPSS Statistics Data Document (.sav) fileMicrosoft Excel Worksheet (.xlsx)FIS File (.fis)
How the data were acquired	Data were collected in two steps:Data collection was performed to prepare the fuzzy system input. To prepare the first set of data, 180 articles from the “interactive system” keyword search in the “Computer Science” category were extracted from the WoS Scientific Database. The second category of data was the opinions of experts. Ten experts in this field were selected by searching the search portal of academic researchers at http://academic.research.microsoft.com and searching in the Google search engine at http://www.google.com. After sending an electronic invitation to these experts, three people accepted the invitation to participate in the study. Experts' acquaintance sessions with the objectives and details of the study were conducted by video conference via Skype. They were then asked to determine the modes according to the five criteria and compare them in the next step. Thus, input data were provided for the multi-factor evaluation method.
Data format	RawAnalyzedFiltered
Description of data collection	We conducted the abstract content analysis for 180 articles to support the following purposes: 1. To investigate the categories of interactive systems in terms of their application, 2. To investigate the most applicable usability evaluation criteria which are applied for the evaluation of the interactive systems, 3. To investigate the most used evaluation criteria for each group of interactive systems. The obtained data were used to design the membership functions and rules of the FIS.In this study, the purpose of designing the interactive system was to measure the impact of usability criteria on the entire fuzzy interactive system. To design this fuzzy interactive system, six classes or factors called business class, game class, urban class, education class, medical class, and military class were considered for the system. Using the fuzzy toolbox in MATLAB, fuzzy membership functions were then designed for each factor based on how the data was distributed. The designed system has one non-fuzzy input, three fuzzy input criteria, and five output criteria. One input was not considered fuzzy as the target of the system. The three fuzzy input metrics included user
	participation, user activity, and information processing. The system is designed based on fuzzy MFs and if-then rules. After completing the design of the interactive system, a FIS Mamdani-based was implemented in the system implementation phase. Data in the implementation of the ASR interactive system was multi-model with four data classification methods that applied one of the AMLMs in each case. The using the data sets in the FIS system leaded to the generation of more than one hundred effective rules. Usability criteria are generally in two groups: fuzzy variables and linguistic variables. After running and implementing the system, the system was tested.
Data source location	Web of Science Core Collectionhttp://academic.research.microsoft.comhttp://www.google.com
Data accessibility [2,3]	**Repository name:** Mendeley Data**Data identification number:** 10.17632/k7mxdhpp34.1**Direct link to the dataset:**https://data.mendeley.com/datasets/k7mxdhpp34/1
Related research article	A. Asemi and A. Asemi. “A Judgment-Based Model for Usability Evaluating of Interactive Systems Using Fuzzy Multi Factors Evaluation (MFE)”. *Applied Soft Computing*. https://doi.org/10.1016/j.asoc.2022.108411.

## Value of the Data

• The Data [1] could be used as a structured source of usability evaluation factors of interactive systems. Usability evaluation is an important research topic in software engineering. Future studies related to usability evaluation directly can select or use the determined evaluation factors from the current method, techniques, and tools.
• Since the evaluation of usability by interactive systems is a decision issue, it has a great impact on the overall improvement of interactive systems. The solutions presented in this paper are an efficient and dynamic evaluation method to improve the evaluation process. Interactive system designers can use this integrated model with three evaluation steps to improve the evaluation process. The proposed model can also be used to evaluate the interactive system, information evaluation and perform complex experimental tests.
• The data and files presented in this study help to classify interactive systems.
• Also, by using this tool and updating key factors in evaluating the usability of interactive systems, a fuzzy inference analyzer system with fixed membership functions can be implemented to formally evaluate interactive systems.
• Also, with the presented methods, the usability factors for each category of interactive systems can be weighted based on fuzzy multi-factor evaluation. The researcher then develops usability evaluation for interactive systems and proposes the evaluation process. This process can be compared to multiple interactive systems.
• Future studies may improve FIS by integrating artificial neural networks.

## 1 Data Description

**MVML criteria Microsoft Visio Document (.vsd) file** related to the process of selection criteria from applied criteria for evaluation of SISs. It shows how the selection of the proper criteria for evaluation of ALAs in disabled children SISs is done by the experts.

**Image 1:**
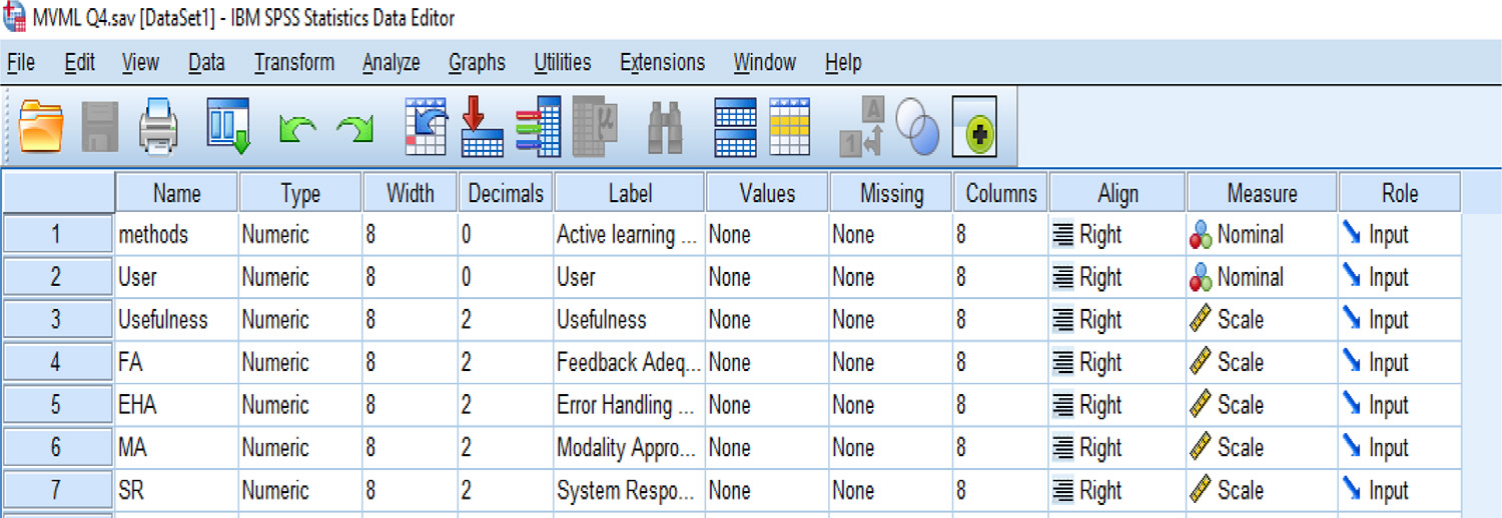
Variable view of data.

**MVML Q4 (SPSS Statistics Data Document (.sav)) file** is related to data which is gathered through usability evaluator system testing process. The data involve the measuring of usability variables for 4 different modes of ASR system while 10 users are using the ASR system. Each user uses the system in all four modes, and we measured the usability variables based on related equations. The variable view of data is shown in Image 1 and Table 1 shows the collected data. (The raw data as a **MVML.xlsx** file attached along with the article as a Supplementary file).

**Table 1:** Collected data for testing of FIS in evaluation of usability factors.

Methods	User	Usefulness	FA	EHA	MA	SR
1	1	70.00	34.00	4.00	4.00	0.00
1	2	60.00	30.00	5.00	3.00	3.00
1	3	80.00	25.00	5.00	5.00	0.00
1	4	77.00	35.00	4.00	4.00	4.00
1	5	63.00	34.00	4.00	4.00	0.00
1	6	75.00	34.00	3.00	4.00	3.00
1	7	65.00	40.00	5.00	3.00	10.00
1	8	70.00	30.00	4.00	4.00	4.00
1	9	55.00	40.00	3.00	3.00	3.00
1	10	85.00	35.00	5.00	5.00	5.00
2	1	21.00	31.00	16.00	5.00	40.00
2	2	15.00	25.00	15.00	5.00	30.00
2	3	25.00	35.00	25.00	15.00	30.00
2	4	30.00	25.00	20.00	5.00	45.00
2	5	10.00	40.00	10.00	10.00	65.00
2	6	20.00	40.00	20.00	10.00	60.00
2	7	15.00	30.00	15.00	15.00	40.00
2	8	10.00	30.00	10.00	10.00	60.00
2	9	35.00	20.00	30.00	10.00	50.00
2	10	22.00	35.00	15.00	10.00	30.00
3	1	12.00	30.00	40.00	60.00	34.00
3	2	5.00	30.00	50.00	60.00	30.00
3	3	15.00	30.00	80.00	75.00	25.00
3	4	15.00	20.00	45.00	63.00	25.00
3	5	10.00	35.00	65.00	60.00	35.00
3	6	10.00	20.00	60.00	65.00	40.00
3	7	15.00	35.00	65.00	70.00	30.00
3	8	15.00	25.00	60.00	50.00	30.00
3	9	20.00	35.00	55.00	70.00	25.00
3	10	10.00	40.00	65.00	60.00	35.00
4	1	4.00	5.00	15.00	30.00	30.00
4	2	5.00	10.00	15.00	35.00	15.00
4	3	5.00	0.00	25.00	25.00	30.00
4	4	6.00	0.00	30.00	30.00	30.00
4	5	6.00	0.00	10.00	10.00	10.00
4	6	8.00	10.00	20.00	20.00	20.00
4	7	5.00	10.00	15.00	15.00	15.00
4	8	8.00	0.00	10.00	15.00	10.00
4	9	6.00	5.00	30.00	35.00	30.00
4	10	6.00	10.00	22.00	25.00	22.00

**MVML Q4 (SPSS Statistics Output Document (.spv)) file** is related to analysis of data in MVML Q4. Sav. (**MVML.xlsx** file is available too). This file includes all the analysis results that have conducted for process of testing.

**Usability Evaluator.fis (FIS File (.fis)) file** is related to structure of usability system evaluator. This file includes all the input and output variables as well as all related membership functions. Images 2–12 show input and output variables and all membership functions. Th membership functions are designed based on distribution of data and variable description by experts.

**Image 2:**
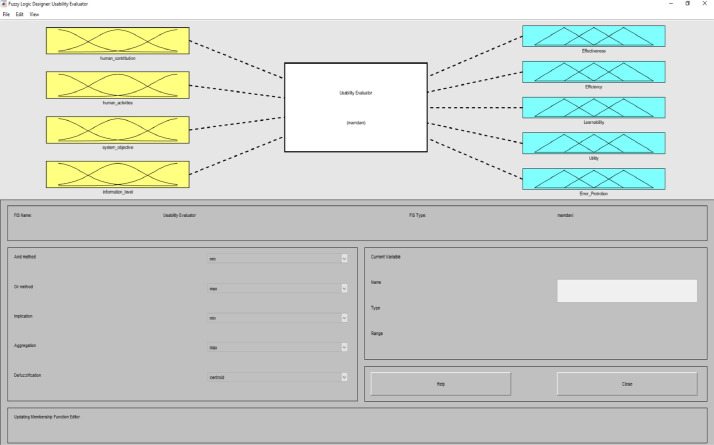
Input and output variables.

**Image 3:**
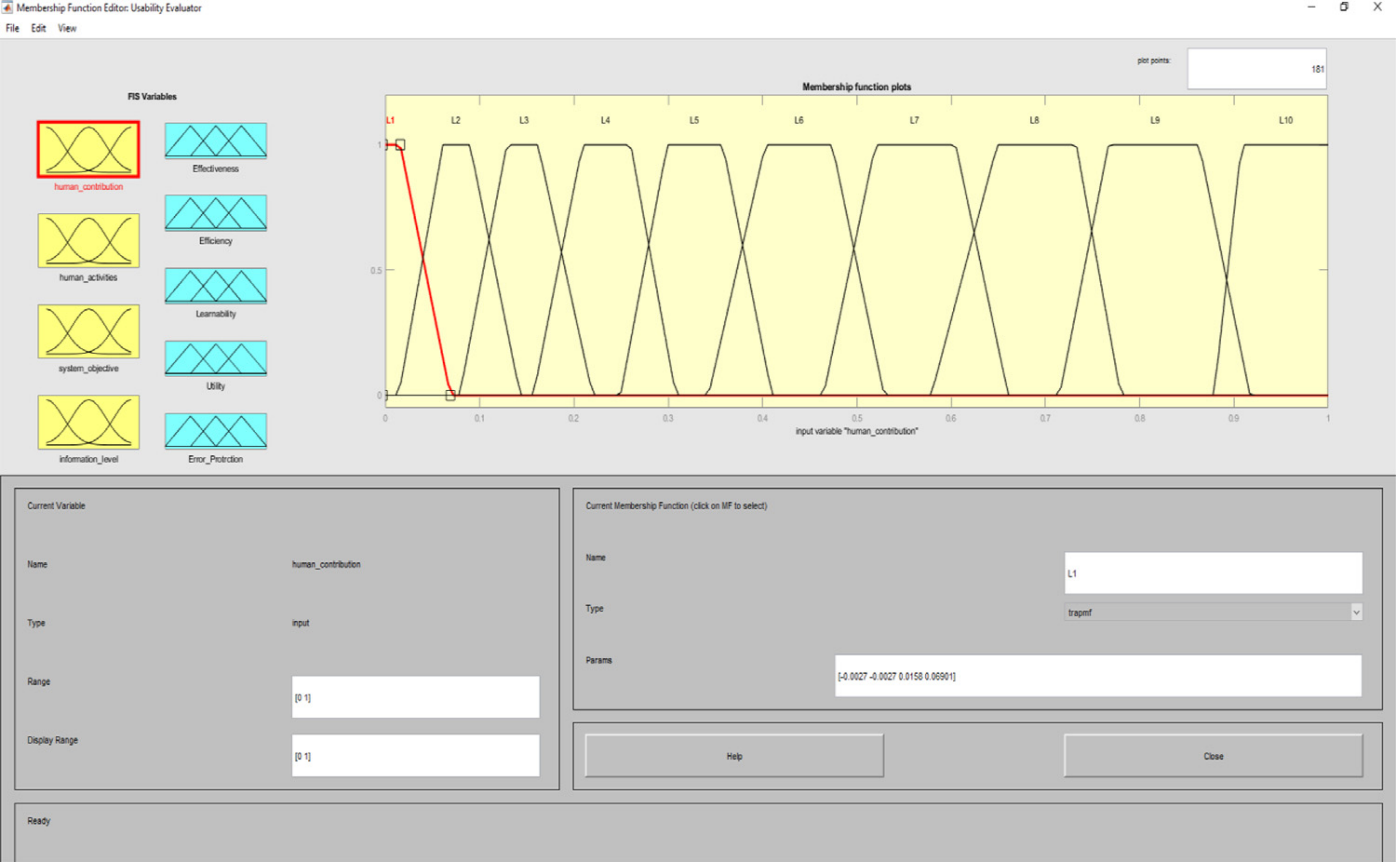
Input 1.

**Image 4:**
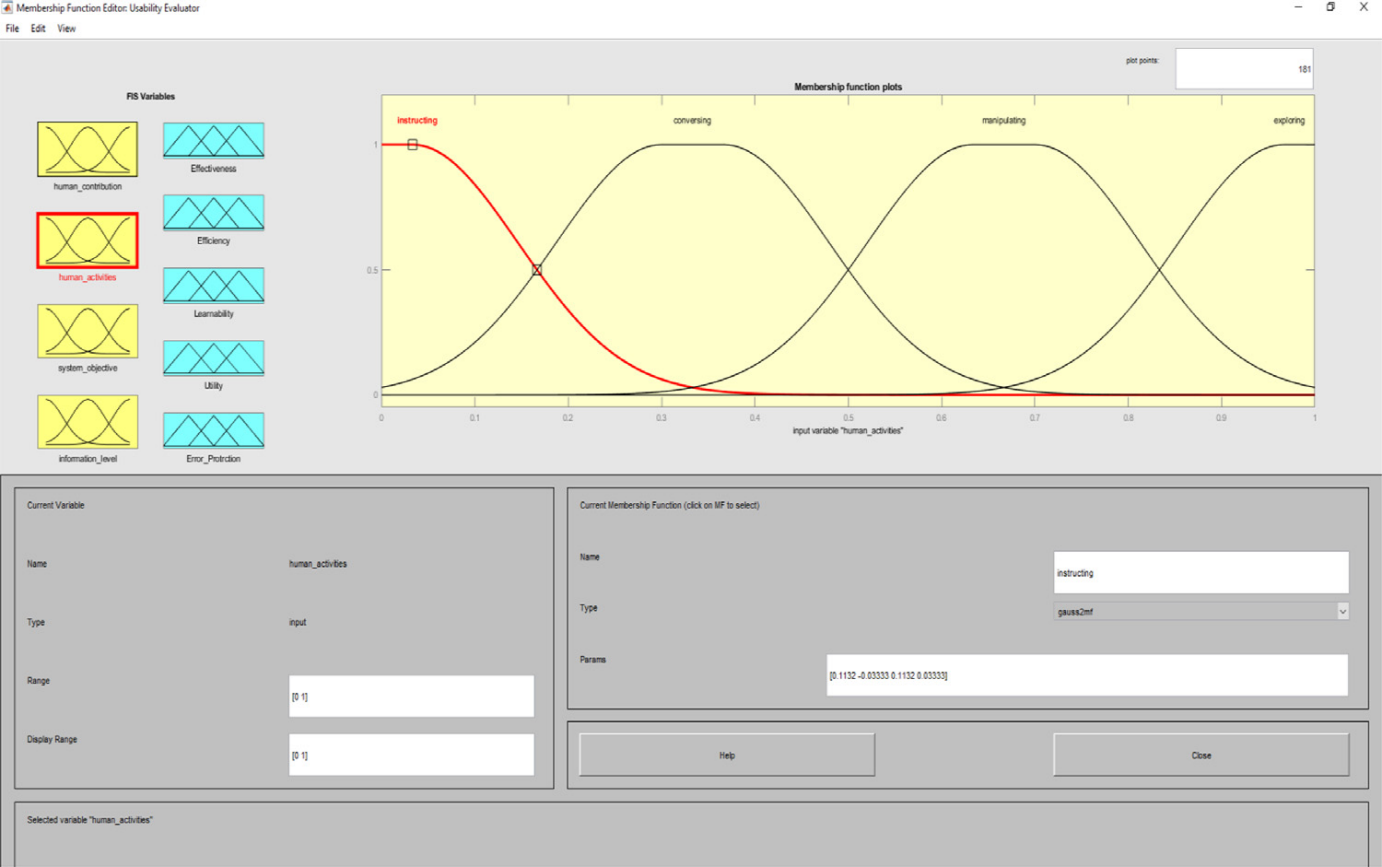
Input 2.

**Image 5:**
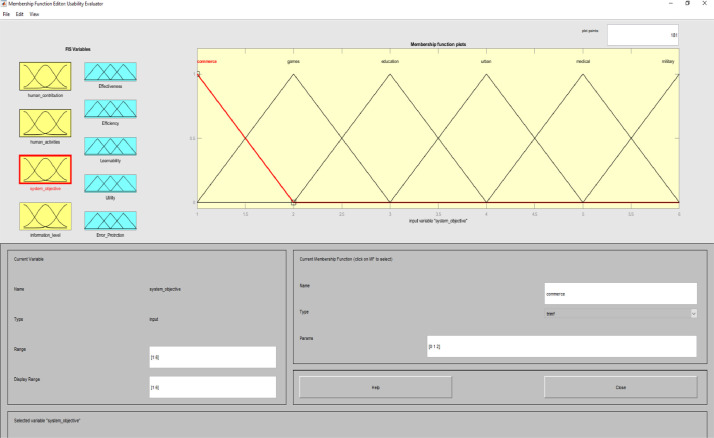
Input 3.

**Image 6:**
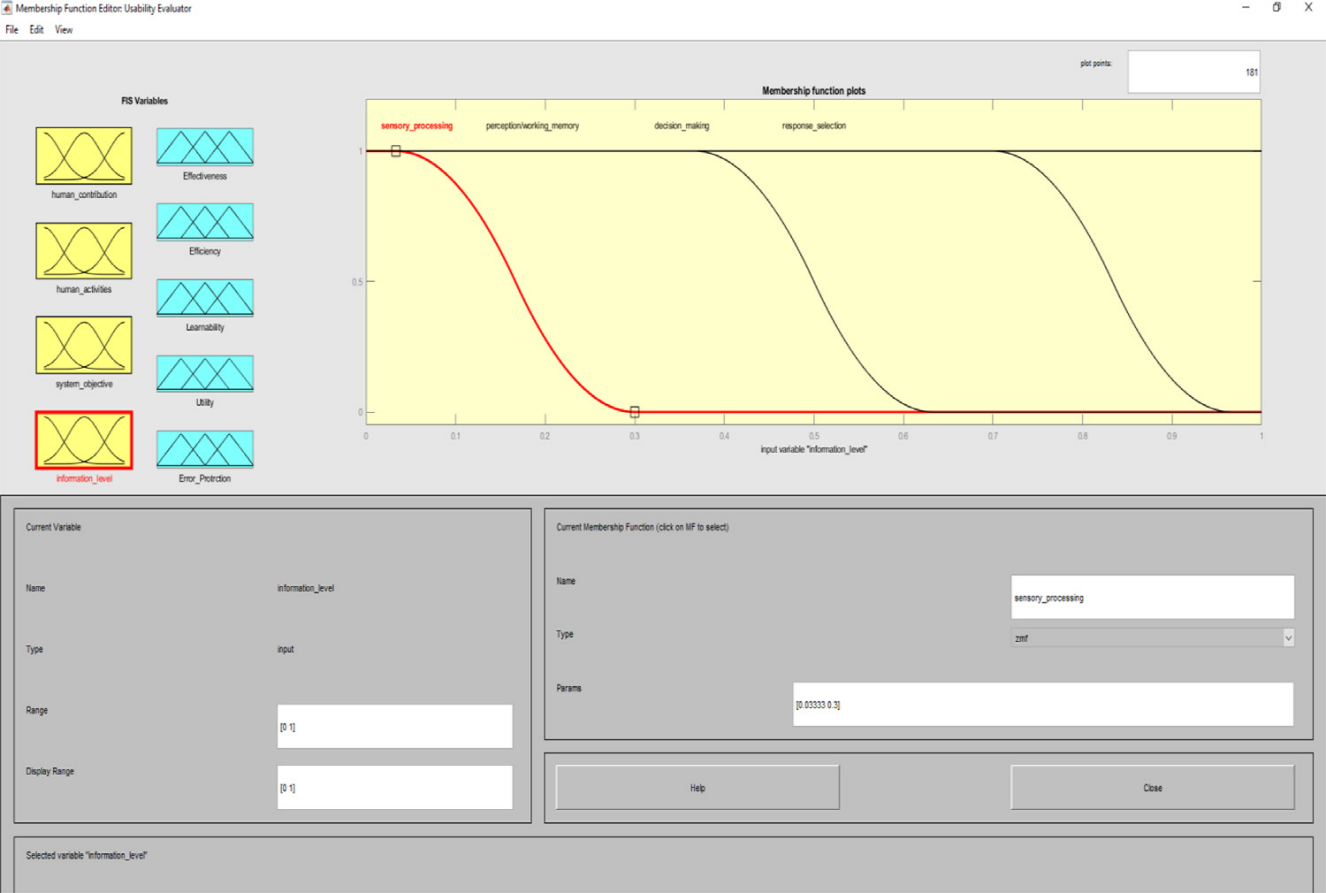
Input 4.

**Image 7:**
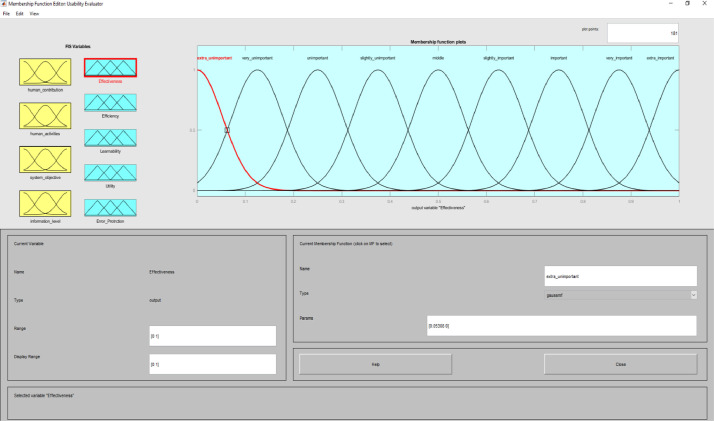
Output 1.

**Image 8:**
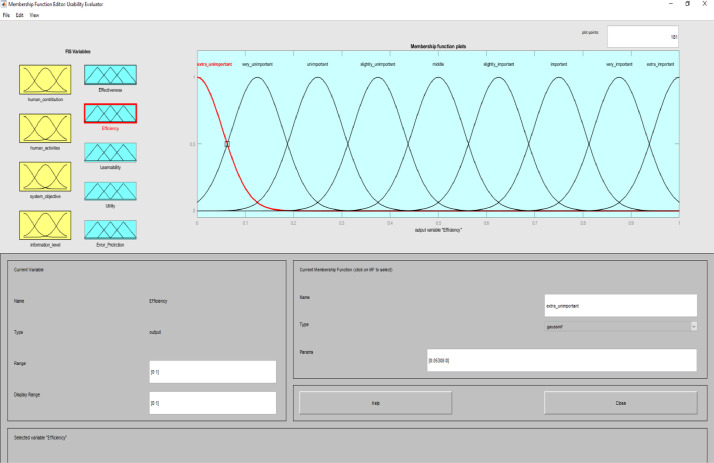
Output 2.

**Image 9:**
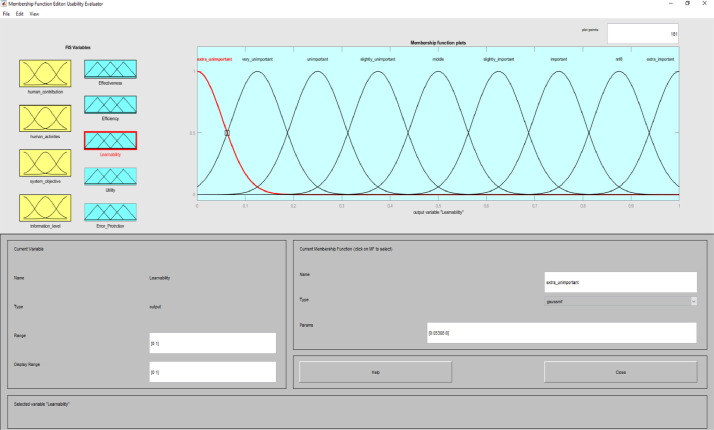
Output 3.

**Image 10:**
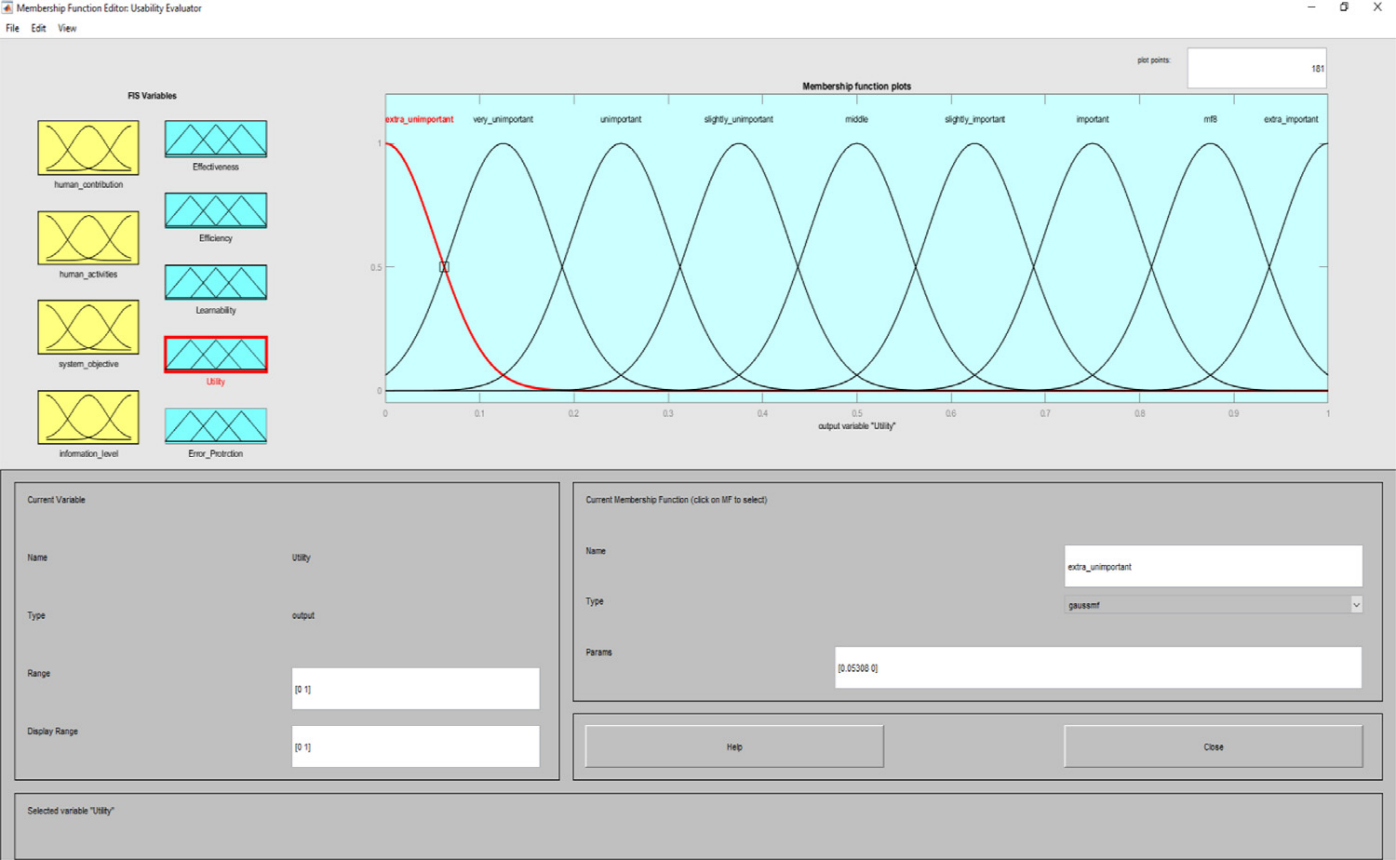
Output 4.

**Image 11:**
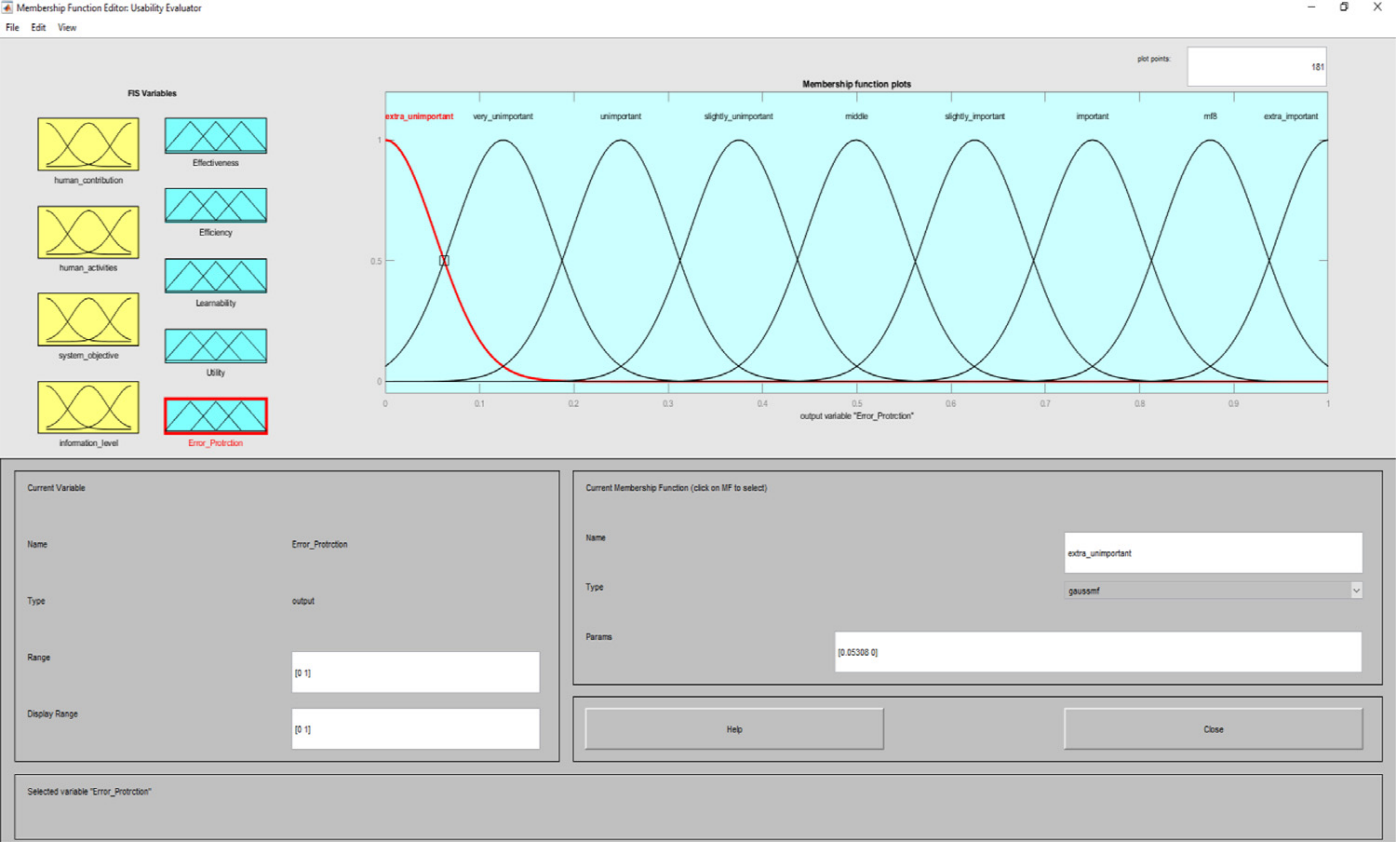
Output 5.

**Image 12:**
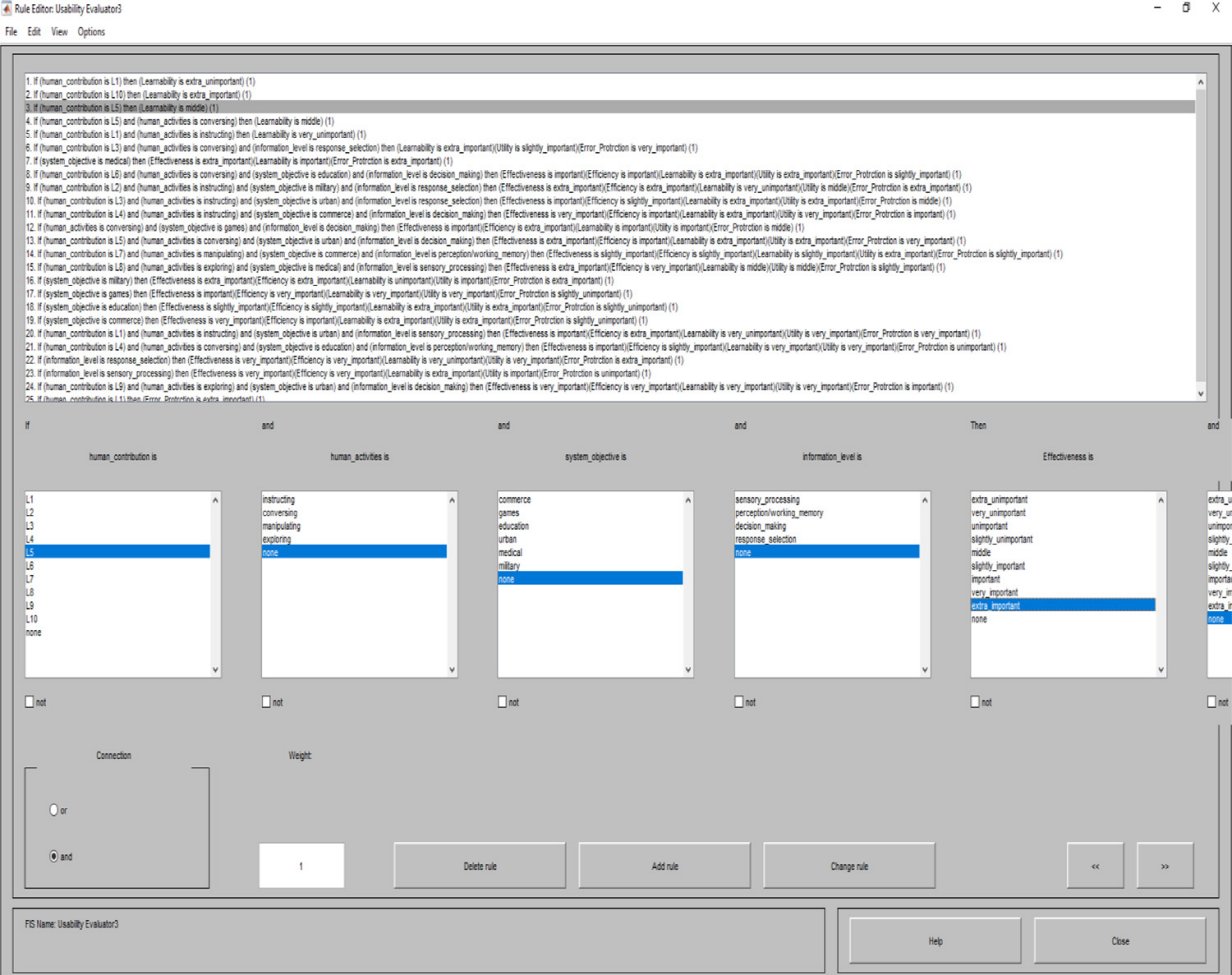
A part of knowledge base in usability evaluator system.

**Usability Evaluator3.fis (Usability Evaluator3.fis) file** is related to fuzzy inference system that we use for evaluating usability factors. This FIS has a knowledge base which is including all the rules based on human knowledge. The rules are presented in the following. The number which is determined in parenthesis at the end of each rule shows the weight of that rule.

**Table:** 

1. If (human_contribution is L1) then (Learnability is extra_unimportant) (1)
2. If (human_contribution is L10) then (Learnability is extra_important) (1)
3. If (human_contribution is L5) then (Learnability is middle) (1)
4. If (human_contribution is L5) and (human_activities is conversing) then (Learnability is middle) (1)
5. If (human_contribution is L1) and (human_activities is instructing) then (Learnability is very_unimportant) (1)
6. If (human_contribution is L3) and (human_activities is conversing) and (information_level is response_selection) then (Learnability is extra_important)(Utility is slightly_important)(Error_Protrction is very_important) (1)
7. If (system_objective is medical) then (Effectiveness is extra_important)(Learnability is important)(Error_Protrction is extra_important) (1)
8. If (human_contribution is L6) and (human_activities is conversing) and (system_objective is education) and (information_level is decision_making) then (Effectiveness is important)(Efficiency is important)(Learnability is extra_important)(Utility is extra_important)(Error_Protrction is slightly_important) (1)
9. If (human_contribution is L2) and (human_activities is instructing) and (system_objective is military) and (information_level is response_selection) then (Effectiveness is extra_important)(Efficiency is extra_important)(Learnability is very_unimportant)(Utility is middle)(Error_Protrction is extra_important) (1)
10. If (human_contribution is L3) and (human_activities is instructing) and (system_objective is urban) and (information_level is response_selection) then (Effectiveness is important)(Efficiency is slightly_important)(Learnability is extra_important)(Utility is extra_important)(Error_Protrction is middle) (1)
11. If (human_contribution is L4) and (human_activities is instructing) and (system_objective is commerce) and (information_level is decision_making) then (Effectiveness is very_important)(Efficiency is important)(Learnability is extra_important)(Utility is very_important)(Error_Protrction is important) (1)
12. If (human_activities is conversing) and (system_objective is games) and (information_level is decision_making) then (Effectiveness is important)(Efficiency is extra_important)(Learnability is important)(Utility is important)(Error_Protrction is middle) (1)
13. If (human_contribution is L5) and (human_activities is conversing) and (system_objective is urban) and (information_level is decision_making) then (Effectiveness is extra_important)(Efficiency is important)(Learnability is extra_important)(Utility is extra_important)(Error_Protrction is very_important) (1)
14. If (human_contribution is L7) and (human_activities is manipulating) and (system_objective is commerce) and (information_level is perception/working_memory) then (Effectiveness is slightly_important)(Efficiency is slightly_important)(Learnability is slightly_important)(Utility is extra_important)(Error_Protrction is slightly_important) (1)
15. If (human_contribution is L8) and (human_activities is exploring) and (system_objective is medical) and (information_level is sensory_processing) then (Effectiveness is extra_important)(Efficiency is very_important)(Learnability is middle)(Utility is middle)(Error_Protrction is slightly_important) (1)
16. If (system_objective is military) then (Effectiveness is extra_important)(Efficiency is extra_important)(Learnability is unimportant)(Utility is important)(Error_Protrction is extra_important) (1)
17. If (system_objective is games) then (Effectiveness is important)(Efficiency is very_important)(Learnability is very_important)(Utility is very_important)(Error_Protrction is slightly_unimportant) (1)
18. If (system_objective is education) then (Effectiveness is slightly_important)(Efficiency is slightly_important)(Learnability is extra_important)(Utility is extra_important)(Error_Protrction is slightly_unimportant) (1)
19. If (system_objective is commerce) then (Effectiveness is very_important)(Efficiency is important)(Learnability is extra_important)(Utility is extra_important)(Error_Protrction is slightly_unimportant) (1)
20. If (human_contribution is L1) and (human_activities is instructing) and (system_objective is urban) and (information_level is sensory_processing) then (Effectiveness is important)(Efficiency is extra_important)(Learnability is very_unimportant)(Utility is very_important)(Error_Protrction is very_important) (1)
21. If (human_contribution is L4) and (human_activities is conversing) and (system_objective is education) and (information_level is perception/working_memory) then (Effectiveness is important)(Efficiency is slightly_important)(Learnability is very_important)(Utility is very_important)(Error_Protrction is unimportant) (1)
22. If (information_level is response_selection) then (Effectiveness is very_important)(Efficiency is very_important)(Learnability is very_unimportant)(Utility is very_important)(Error_Protrction is extra_important) (1)
23. If (information_level is sensory_processing) then (Effectiveness is very_important)(Efficiency is very_important)(Learnability is extra_important)(Utility is important)(Error_Protrction is unimportant) (1)
24. If (human_contribution is L9) and (human_activities is exploring) and (system_objective is urban) and (information_level is decision_making) then (Effectiveness is very_important)(Efficiency is very_important)(Learnability is very_important)(Utility is very_important)(Error_Protrction is important) (1)
25. If (human_contribution is L1) then (Error_Protrction is extra_important) (1)

## 2 Experimental Design, Materials and Methods

The data presented in this paper are proposed to provide a model for evaluating computer interactive systems based on expert judgments. This model is suitable for situations where an experimental evaluation of an interactive system is costly or complex. This model is proposed for automation of usability evaluation of interactive systems in main three steps. In first step we collected the evaluation criteria from literature review through factor analysis. In details first step pre-process of evaluation included classify interactive systems, determination of usability criteria, implementation of a fuzzy inference analyser, and formulation of usability evaluation. In second step, the fuzzy multi-Criteria decision-making approach is applied for ranking of factors. In details second step included usability evaluating of an interactive system, Identify the interactive system class, set related evaluation formula, judgment sampling, and assess factors based on Fuzzy calculation of distance. In third step, a fuzz fuzzy inference system is designed and implemented for evaluation of interactive systems. In details third step included Usability comparison between more than one interactive system, Identify the interactive system class, pairwise comparison of systems on related criteria based on fuzzy variables, and obtain eigen vector. Finally, we implemented four interactive systems. The usability evaluation conducted for systems through traditional evaluation methods and proposed method. The results are compared and analyzed through statistical analysis methods.

In this model, four main HCI classifications are considered, including human participation, human activities, system purpose, and information processing. Sheridan & Verplank's method is used to determine the level of human participation [9]. They considered ten levels for human participation. These ten levels included 1. At this level human has no participation and the computer is the decision-maker for everything. Here, the computer operates independently and ignores the human, 2. At this level, only the human is informed about the computer's decision, 3. At this level, the human is informed by the computer if requested. 4. At this level, the computer operates automatically and notifies humans if necessary, 5. At this level the computer allows humans to have a limited time to veto before running automatically, 6. In this Level, If a human approves, the computer executes the command, 7. At this level, the computer suggests an alternative, 8. At this level, the computer limits the choice to a few decision options, or 9. The computer offers a complete set of decision options, or 10. The computer offers no choice and man has to make all the decisions.

In implementing the expert system of this study, we considered these ten levels as factors in determining the type of interactive system. For human activities, user actions were considered. These activities include training, conversation, manipulation and navigation, and exploration [11]. The purpose of the system was determined based on the purpose of the interactive systems and the usability assessment is directly affected by the purpose of the interactive system. In information processing, the performance of different levels overlaps at the same time. Levels of information processing in interactive systems are included sensory processing, perception/working memory, decision making, and response selection.

To determine usability criteria, two sources of usability assessment criteria were considered ISO standards, and literature review. Next, to implement the fuzzy inference analyzer, a fuzzy control strategy was used to plot the given inputs through the rules, and generate the output based on these rules. In this inferential system, inputs were included four classes of interactive systems and outputs were included five capabilities criteria. Of course, it should be noted that one of the inputs (system purpose) is not considered as a fuzzy variable because we only define one main purpose for an interactive system. The other three inputs and the five outputs are considered fuzzy variables.

MATLAB software with fuzzy logic toolbox was used to implement the fuzzy inference system (FIS). This system was implemented based on Mamdani. It is noteworthy that this system was used to measure the impact of usability criteria on the entire interactive system. The following is a fuzzy control strategy for drawing given inputs through rules, and generating output based on these rules. Expert judgment is then used to control the rules and manipulate the rules. These experts judge on the interactive system and present the input of the fuzzy system based on their expert opinions. In this method, the necessary data for the multi-factor evaluation method are presented and the evaluation of the criteria is performed based on the fuzzy calculation distance. Finally, usability is compared to more than one interactive system. An experimental example is provided in the main paper and all the equations are in the main paper.

## Ethics Statements

Our work didn't involve human subjects

Our work didn't involve animal experiments

Our work didn't involve data collected from social media platforms

## CRediT Author Statement

Authors' contribution is equal for all sections of the paper.

## Declaration of Competing Interest

The authors declare that they have no known competing financial interests or personal relationships that could have appeared to influence the work reported in this paper.

## Data Availability

Data for Usability Evaluating of Interactive Systems based on the Judgment-Based Model (Original data) (Mendeley Data).
