# Corrigendum to ‘Data to evaluate the usability of an interactive system based on a judgment-based model’ [Data in Brief 43 (2022) 1–14/ 108418]

**DOI:** 10.1016/j.dib.2022.108472

**Published:** 2022-07-15

**Authors:** Adeleh Asemi, Asefeh Asemi

**Affiliations:** a Department of Software Engineering, Faculty of Computer Science and Information Technology, Universiti Malaya, 50603 Kuala Lumpur, Malaysia; b Doctoral School of Economics, Business, & Informatics, Corvinus University of Budapest, 1093 Budapest, Fovam ter 8., Hungary

The authors regret ‘Please change the acknowledgment to: The present publication is the outcome of the project “Project no. TKP2020-NKA-02 has been implemented with the support provided from the National Research, Development and Innovation Fund of Hungary, financed under the Tématerületi Kiválósági Program funding scheme.”.

The authors would like to apologise for any inconvenience caused.

## Declaration of Competing Interest

The authors declare that they have no known competing financial interests or personal relationships that could have appeared to influence the work reported in this paper.

